# Vascular–immune zonation sets organ-specific metastatic checkpoints

**DOI:** 10.3389/fimmu.2026.1944407

**Published:** 2026-08-25

**Authors:** Ziheng Zheng, Minqian Ou, Yuedong Chen, Deyong Gan

**Affiliations:** 1 The First Clinical College of Chongqing Medical University, Chongqing, China; 2 Department of Gastrointestinal Surgery, Tongliang Hospital of the First Affiliated Hospital of Chongqing Medical University, Chongqing, China

**Keywords:** endothelial heterogeneity, immune checkpoint blockade, metastatic niche, spatial transcriptomics, tissue-resident immunity, tumor dormancy

## Abstract

Organ-specific metastatic niches begin at vascular–immune interfaces, where endothelial heterogeneity and tissue-resident immunity shape the fate of disseminated tumor cells. Hemodynamics and vascular geometry shape initial tumor-cell encounters, whereas endothelial and perivascular immune states determine whether arrest progresses to extravasation, immune elimination, dormancy, or outgrowth. This Mini Review uses vascular–immune zonation as an organizing framework for comparing four common metastatic sites. In the lung, capillary endothelium, alveolar macrophages, and rapidly recruited neutrophils form a responsive barrier that can switch from tumor control to metastatic support. Liver sinusoids couple scavenging and immune tolerance, allowing Kupffer cells and recruited macrophages to exert stage-dependent effects that can also suppress systemic immunotherapy. At the blood–brain barrier, endothelial and mural-cell states interact with microglia and astrocytes to control entry and adaptation. In bone, specialized vessels overlap with hematopoietic and osteogenic niches that metastatic cells exploit for immune evasion and dormancy escape. Across organs, the same immune-cell label does not imply the same function because location, ontogeny, and neighboring stromal cells constrain cell state. Therapeutic design should therefore target pathogenic microdomains and transition states rather than deplete a cell class throughout the body. Spatially matched sampling of metastatic lesions, adjacent tissue, and blood will be needed to translate this site-aware model into biomarkers and combination therapies.

## 1 Introduction

The distribution of metastases is non-random. Paget’s seed-and-soil hypothesis supplied the enduring biological premise, while work on pre-metastatic niches showed that distant tissues can be altered before disseminated tumor cells arrive (1, 2). Organotropism reflects several filters, including circulation, tumor-intrinsic traits, extracellular vesicles, extracellular matrix, and local stromal and immune programs (3). Tumor-derived exosomal integrins can direct vesicles to particular organs, providing experimental evidence that distant vascular and stromal cells receive organ-selective signals (4). The immune component of this process is equally site dependent: resident cells, recruited myeloid populations, and tissue-specific constraints can either remove disseminated cells or permit their persistence (5, 6).

Recent syntheses describe metastatic organotropism, tissue-specific cancer immunity, endothelial remodeling, and macrophage diversity (7–10). For cross-organ comparison, vascular entry and immune remodeling are best considered as a coupled spatial process. Endothelial cells control adhesion and permeability while presenting chemokines and immune-regulatory ligands; perivascular immune cells, in turn, alter barrier function and tumor-cell fate. We use vascular–immune zonation to mean the spatial patterning of these coupled states. The term includes classical anatomical zonation, as in the liver, and smaller inducible microdomains at capillary, sinusoidal, neurovascular, or marrow interfaces. This framework asks a focused question: which local vascular–immune states decide whether a disseminated tumor cell is cleared, held dormant, or allowed to grow?

## 2 Vascular–immune microdomains as metastatic checkpoints

The pre-metastatic niche concept began with the observation that vascular endothelial growth factor receptor 1-positive bone-marrow progenitors cluster at future metastatic sites (11). Subsequent work has placed those recruited cells within perivascular niches that regulate invasion, stemness, immune escape, and dormancy (12). Endothelial cells are not interchangeable conduits. Single-cell studies reveal organ- and vessel-segment-specific endothelial programs, collectively termed angiodiversity (13). Multi-organ atlases likewise show that stromal and immune identities retain strong tissue signatures even when they share conventional markers (14, 15). Thus, a CD68-positive macrophage or PECAM1-positive endothelial cell cannot be assigned a fixed metastatic function without knowing its origin and location.

**Figure 1:**
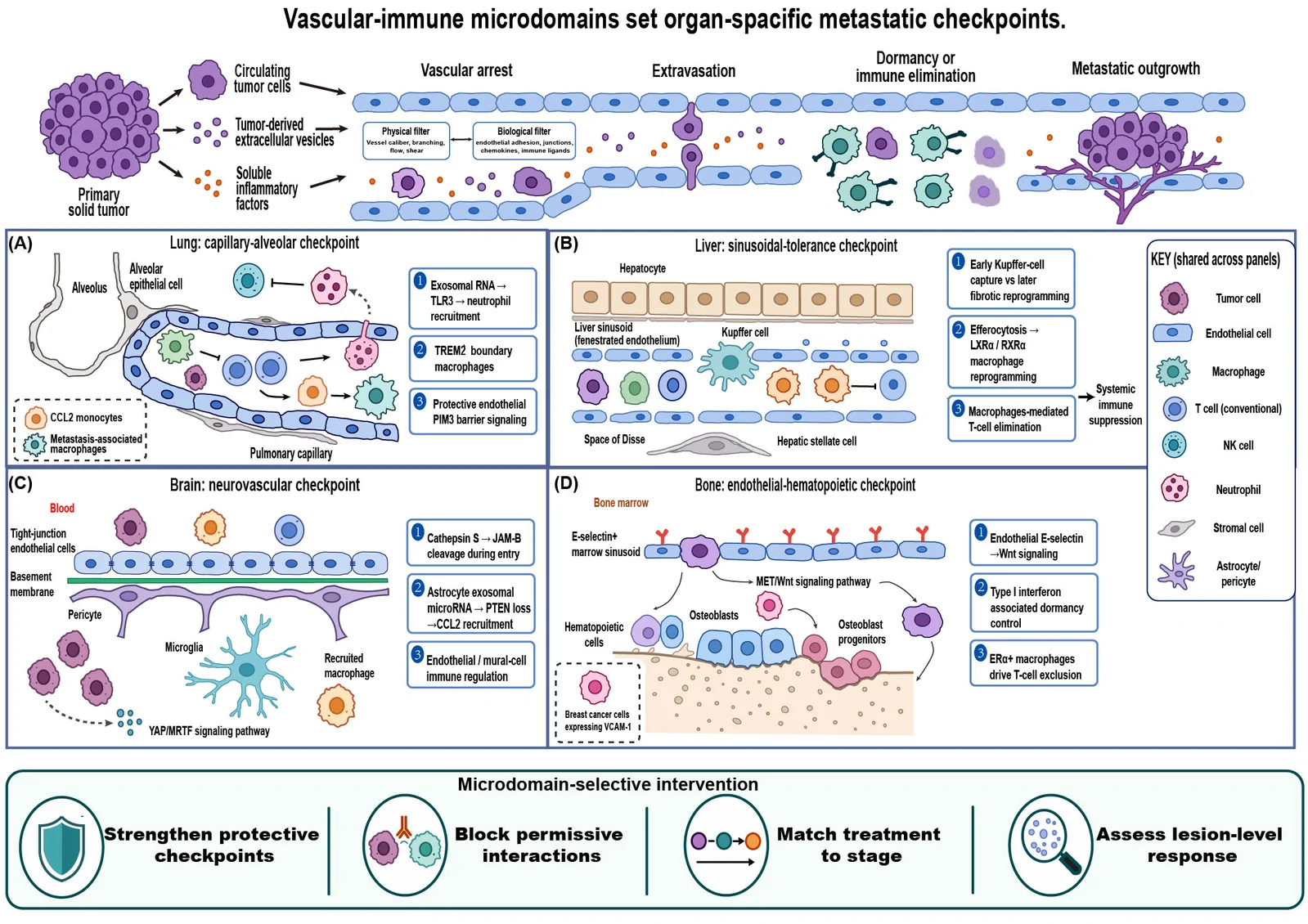
Vascular–immune microdomains set organ-specific metastatic checkpoints. Tumor-derived extracellular vesicles and soluble factors condition distant vascular interfaces before or during the arrival of circulating tumor cells. Organ-specific vascular geometry, vessel caliber, flow, and shear form a physical filter for initial tumor-cell contact or mechanical confinement. Endothelial adhesion, junctional, chemokine, and immune-regulatory programs form a coupled biological filter that determines whether transient contact progresses to stable arrest, extravasation, leukocyte recruitment, immune clearance, or colonization. **(A)** The pulmonary capillary–alveolar interface can recruit monocytes and neutrophils yet preserve barrier function. **(B)** In liver sinusoids, Kupffer-cell scavenging can restrict early seeding, whereas fibrosis, efferocytosis-driven macrophage reprogramming, and T-cell elimination support established metastases and may suppress systemic immunity. **(C)** The brain neurovascular unit controls entry through tight junctions and mural cells; astrocytes and central nervous system-native myeloid cells then shape perivascular adaptation and immune exclusion. **(D)** In bone marrow, E-selectin-positive sinusoids promote bone colonization, while metastatic cells also co-opt hematopoietic and osteogenic niches through osteoclast recruitment, interferon-dependent dormancy control. The bottom strip distinguishes strengthening protective checkpoints, including vascular normalization or barrier preservation, from disrupting permissive cellular interactions; treatment should be matched to metastatic stage and assessed at the lesion level. Solid arrows indicate experimentally supported directionality; dashed arrows indicate context-dependent or proposed relationships; blunt-ended lines denote inhibition.

Four linked parts are initiated or modulated at and around vascular interfaces (**Figure 1**). Initial arrest is not determined by either hemodynamics or endothelial identity alone. Vessel caliber, branching geometry, flow velocity, and shear govern where circulating tumor cells make prolonged contact or become mechanically confined, whereas endothelial adhesion molecules, chemokines, junctional state, and immune-regulatory ligands determine whether transient contact progresses to stable arrest, extravasation, or clearance (16). These variables also interact because shear influences tumor-cell adhesion and endothelial signaling. Endothelial phenotypes more directly recruit and position than fully activate immune cells: normalized or high-endothelial-venule-like vessels can support lymphocyte entry, whereas tumor-conditioned endothelial programs may restrict T-cell trafficking or favor myeloid accumulation (17–19). Subsequent activation is completed by antigen-presenting and stromal cues within the local microdomain. Resident macrophages, natural killer cells, and T cells then determine whether newly arrived cells are eliminated or enter a protected state, while recruited myeloid cells, vascular remodeling, and tissue-repair programs can release surviving cells from dormancy. Macrophage enhancer programs are imposed jointly by ontogeny and local signals, explaining why apparently similar cells behave differently across organs (20, 21). Endothelial state is therefore bidirectional: vessel normalization can improve leukocyte entry and immunostimulation, whereas indiscriminate vascular inhibition may damage a protective barrier (17). Endothelial immune-checkpoint and chemokine programs belong in the same therapeutic model as leukocyte targets (18).

Regarding these decisions as a spatial sequence prevents two common interpretive errors. Cell abundance is not a proxy for function: an expanded myeloid population may eliminate tumor cells, suppress lymphocytes, or support tissue repair depending on its state and position. Nor is an induced program intrinsically protective or pathogenic. Interferon-responsive endothelium can recruit leukocytes but can also express inhibitory ligands, while macrophage efferocytosis repairs injured tissue yet may silence antitumor T cells. The informative comparison is therefore not simply organ A versus organ B. It is baseline tissue identity versus a tumor-induced state, followed across early seeding, dormant persistence, and macroscopic growth. Dissociation-based atlases define candidate populations; spatial adjacency to a vessel segment or metastatic cell is needed to assign checkpoint function (13, 15, 18).

## 3 Lung: the capillary–alveolar checkpoint

The pulmonary circulation exposes circulating tumor cells to high-throughput flow through an extensive, narrow capillary network; this geometry increases mechanical encounters, whereas capillary endothelial junctions and adhesion programs determine whether lodging becomes stable seeding. Primary-tumor hypoxia can recruit suppressive CD11b-positive myeloid cells to the lung and reduce natural-killer-cell cytotoxicity before overt metastasis (22). Alveolar macrophages suppress antitumor T-cell responses in experimental pre-metastatic lungs (23), while recruited neutrophils support breast-cancer-initiating cells (24). Interleukin-17-producing γδ T cells can amplify this neutrophil response, and CCL2-dependent inflammatory monocytes mature into metastasis-associated macrophages (25, 26). The pulmonary checkpoint is therefore a cellular relay, not a neutrophil-only niche.

Lung-resident non-immune cells help initiate the relay. Tumor exosomal RNAs activate Toll-like receptor 3 in alveolar epithelial cells, inducing chemokines that recruit neutrophils (27). Local oxygen sensing can suppress T-cell activity and establish tolerance (28). Inflammation then changes the fate of cells that have already survived dissemination: neutrophil extracellular traps cleave extracellular matrix components and awaken dormant tumor cells, while oxidized lipids and lipid-loaded neutrophils provide additional reactivation and metabolic support (29–31). Spatial mapping has identified TREM2-positive macrophages at the metastatic boundary, supporting the study of geographically restricted cell communities rather than isolated cell types (32). Endothelial responses can also remain protective. A reactive pulmonary capillary endothelial state marked by PIM3 reinforces junctions; PIM inhibition increased leakage and metastatic colonization in experimental models (33). This finding cautions against assuming that every activated endothelial program should be blocked.

Neutrophils make the interpretive problem especially clear. Across models they help establish a permissive lung and awaken dormant cells, but these endpoints occur at different times and need not reflect one stable neutrophil state (24, 29–31). The lineage label should be paired with the recruiting signal, anatomical compartment, activation product, and metastatic stage. Tumor-conditioned neutrophils in capillaries, interstitium, and airspaces encounter different oxygen, lipid, and stromal cues; self-maintaining alveolar macrophages must likewise be distinguished from recruited monocyte descendants (23, 26). This distinction also explains why perturbations applied at different metastatic stages can yield different outcomes.

## 4 Liver: sinusoidal zonation and systemic tolerance

Slow sinusoidal transit and fenestrated liver sinusoidal endothelium prolong contact among circulating tumor cells, Kupffer cells, and an organ constitutively exposed to gut-derived antigens. Kupffer cells are positioned to capture circulating cells and can restrict early metastatic seeding (34, 35). The same tissue-repair and tolerance machinery can later be redirected. Pancreatic-cancer exosomes are taken up by Kupffer cells and promote a fibronectin-rich, myeloid-recruiting niche, while macrophage-derived granulin activates hepatic stellate cells and fibrosis (36, 37). These stage-dependent effects make global macrophage-depletion experiments difficult to interpret as evidence for a single macrophage function.

Physiological liver zonation supplies the spatial template. Commensal signals create periportal immune zones, and spatial proteogenomics has resolved conserved macrophage niches associated with distinct stromal cells (38, 39). Human colorectal liver metastases preserve marked myeloid heterogeneity: macrophage morphology correlates with transcriptional state and prognosis, single-cell studies identify changing immune programs over metastatic progression, and GPNMB-positive mononuclear phagocytes occupy clinically relevant states (40–42). Tissue injury adds another route to convergence. In pancreatic-cancer liver metastasis, macrophage efferocytosis of dead parenchymal cells activates a progranulin–LXRα/RXRα repair program, weakens CD8-positive T-cell function, and supports colonization (43).

The apparent reversal in Kupffer-cell function is not necessarily contradictory. Early intravascular capture, pre-metastatic conditioning, and growth of an established lesion are different biological tests (34–36). A resident macrophage that removes an isolated disseminated cell can later participate in a repair circuit after hepatocyte injury, while recruited monocyte-derived cells add further heterogeneity (37, 39, 43).

The hepatic checkpoint also has systemic consequences. Liver metastases can induce macrophage-mediated deletion of antigen-specific T cells and reduce checkpoint-blockade efficacy (44). A complementary model links hepatic regulatory T cells to systemic suppression of antitumor immunity (45). These mechanisms help explain why a liver lesion may influence responses outside the liver, although their magnitude varies by tumor type and treatment context. Endothelial state may further stratify risk: single-cell analysis of uveal-melanoma liver metastasis reported a senescent endothelial program associated with metastatic support (46). Together, these data favor interventions that reprogram a defined sinusoidal–macrophage unit over organ-wide macrophage or endothelial ablation.

## 5 Brain: the neurovascular checkpoint

Unlike fenestrated liver sinusoids, continuous tight-junction endothelium at the blood–brain barrier makes selective adhesion and barrier remodeling, rather than mechanical lodging alone, critical for brain colonization; surviving cells must then adapt to a neural immune environment (47). Macrophage- and tumor-derived cathepsin S promotes transmigration by cleaving junctional adhesion molecule B, directly connecting a myeloid protease to barrier entry (48). Beyond the vessel wall, reactive astrocytes and microglia accumulate around metastases (49). Microglia can promote breast-cancer colonization through Wnt signaling, and astrocyte-derived exosomal microRNAs reversibly suppress PTEN in metastatic cells, increasing CCL2-dependent myeloid recruitment (50, 51). The neurovascular checkpoint therefore continues after extravasation.

Resident and recruited myeloid cells are not equivalent in brain metastases. Central nervous system-native myeloid cells can drive CXCL10-dependent immune suppression (52), and experimental treatment alters metastasis-associated myeloid states over time (53). Human single-cell studies distinguish disease-specific macrophage and lymphocyte programs across brain tumors and show that incoming leukocytes are instructed by the local tumor environment (54, 55). Spatial profiling further reveals immune neighborhoods that are missed when lesions are dissociated (56). Most directly, single-cell analysis of endothelial and mural cells in brain metastasis identified vascular subtypes with immune-regulatory programs and candidate therapeutic liabilities (57).

The blood–brain barrier should therefore not be treated as a binary gate that is either intact or absent. Entry of an isolated cell, survival of a perivascular micrometastasis, and perfusion of a large lesion involve different endothelial and mural-cell states (47, 57). Even within one metastasis, a remodeled vessel need not have the same permeability, adhesion, or immune-regulatory program as its neighbor. Contrast enhancement or systemic drug exposure may consequently be an incomplete surrogate for the local vascular checkpoint. This heterogeneity helps explain why improving delivery and reversing immune exclusion are related goals but not interchangeable ones.

Physical adaptation and immunity again meet at the vessel. Disseminated cells can spread along abluminal vessels in a pericyte-like state and activate YAP/MRTF signaling (58). Experimental vessel occlusion also produces a hypoxic–ischemic response involving angiopoietin 2 and vascular endothelial growth factor; combined inhibition reduced metastatic outgrowth in model systems (59). These observations argue for stage-specific treatment: preventing entry, reversing established perivascular immune suppression, and restoring drug delivery are related but distinct objectives.

## 6 Bone: endothelial–hematopoietic niche hijacking

Bone metastasis develops within marrow niches that already regulate hematopoietic stem cells, immune tolerance, osteogenesis, and remodeling (60–62). Marrow sinusoids provide a molecular docking environment in which endothelial E-selectin adds tissue-selective signaling to any contribution from local vascular transit. Direct causal evidence implicates marrow endothelium: vascular E-selectin engages tumor cells and activates mesenchymal–epithelial transition and Wnt programs that promote bone colonization, while loss of host E-selectin preferentially reduces bone rather than lung metastasis in experimental models (61). Tumor-intrinsic bone-tropic programs determine access to this environment (63), but local cell contacts govern what follows. Vascular cell adhesion molecule 1 on indolent breast-cancer cells recruits α4β1-positive osteoclast progenitors and drives osteolytic expansion (64). Loss of tumor-cell IRF7 signaling permits immune escape, whereas tumor-intrinsic type I interferon signaling can restrain prostate-cancer outgrowth and maintain dormancy (65, 66). These results place immune surveillance beside the familiar osteoblast–osteoclast “vicious cycle”.

Bone also exposes a conceptual difficulty: dormancy and immune-mediated elimination can look similar in a short experiment. A stable burden of non-proliferating cells may reflect continued immune restraint, adaptation to a hematopoietic niche, or a balance between cell loss and replacement (62, 65, 66). Osteoclast activation can release matrix-derived growth signals while changing which myeloid cells occupy the niche (60, 64). Conversely, a treatment that reduces osteolysis may not have removed dormant tumor cells. Studies should therefore measure tumor-cell state, bone remodeling, and local immunity together and should follow relapse after the perturbation is withdrawn.

Myeloid-cell origin and position refine the model. Monocyte-derived macrophages promote breast-cancer outgrowth in bone (67), and single-cell analysis of human prostate-cancer bone metastases has identified an immunosuppressive microenvironment with actionable myeloid and lymphoid programs (68). Two recent mechanistic studies move beyond correlation. Unbiased proximity tagging found estrogen-receptor-α-positive macrophages that exclude T cells from bone metastatic niches; macrophage-specific loss of this pathway reduced colonization (69). A separate study described iron-transporting macrophages that supply metastatic cells and contribute to cancer-associated anemia (70). Both findings support microdomain-selective intervention, although their generality across tumor types remains to be tested.

## 7 Therapeutic implications

Organ-specific metastatic checkpoints may be strengthened when they are protective and disrupted when they are permissive. Protective strategies include vessel normalization to restore leukocyte entry and preservation or reinforcement of PIM3-dependent pulmonary barrier integrity (17, 33). In contrast, permissive interactions could be exploited by blocking liver exosome uptake, cathepsin-S-mediated blood–brain barrier passage, CCL2-dependent monocyte recruitment, or estrogen-receptor-α-positive macrophage-mediated T-cell exclusion in bone (26, 36, 48, 69). The relevant target is therefore a stage- and site-defined cellular interaction rather than an entire endothelial or immune lineage. Blanket vascular inhibition or systemic macrophage depletion may remove protective as well as pathogenic states.

Translation also requires trial designs that preserve organ information. Baseline and on-treatment biopsies should be annotated by lesion site and prior local therapy; imaging endpoints should be reported for individual lesions as well as the whole patient. Prospective studies can then test three falsifiable predictions of the framework: phenotypically similar immune populations will differ in function across vascular compartments; and a lesion-level vascular–immune signature will predict response better than the corresponding blood measurement.

## 8 Discussion

Vascular–immune zonation offers a practical explanation for site-dependent metastatic behavior: disseminated cells do not encounter “the immune system” in the abstract. They encounter a specific endothelial segment, resident immune population, stromal neighbor, and physiological task. The lung prioritizes gas exchange and rapid inflammatory recruitment; the liver couples filtration with tolerance; the brain protects a tightly regulated neural compartment; and marrow vessels maintain hematopoiesis and bone turnover. Tumors exploit these normal functions, often through tissue-repair programs that are beneficial outside cancer.

The evidence has limits. Much of the causal work uses experimental metastasis, which bypasses early dissemination and may deliver non-physiological tumor-cell numbers. Human spatial studies are usually cross-sectional and often compare unmatched primary and metastatic samples. “Zonation” is rigorously mapped in normal liver but remains an operational description of inducible microdomains in lung, brain, and bone. Treatment, lesion size, primary-tumor lineage, and sampling method can each alter the observed immune state. Moreover, most studies do not manipulate local flow and endothelial identity independently in the same tumor clone, so their relative causal contributions cannot yet be ranked across organs. Direct cross-organ studies using the same tumor clone and matched analytical platform remain uncommon.

Several disputes remain open. It is not known whether different organs converge on a small set of reusable vascular–immune transition states or whether each niche is largely idiosyncratic. The relative contribution of tissue-resident cells and continuously recruited cells also changes with tumor type and treatment, yet most human datasets provide only a snapshot. Finally, local niche reprogramming may alter systemic immunity, as liver-metastasis studies suggest, but the direction and durability of that effect have not been established across cancers (44, 45). These are questions that require matched, longitudinal, multi-organ sampling rather than larger single-site atlases alone.

The strongest next experiments will therefore combine lineage tracing, intravital imaging, spatial multi-omics, and perturbation of defined cell–cell contacts. They should separate niche initiation from established metastasis and report both local and systemic immune effects. Such studies can test whether vascular–immune microdomains predict lesion-level therapy response and whether selective reprogramming preserves physiological tissue defense. We propose that the most informative therapeutic unit may be a pathogenic cellular interaction defined by anatomical site and metastatic stage.
